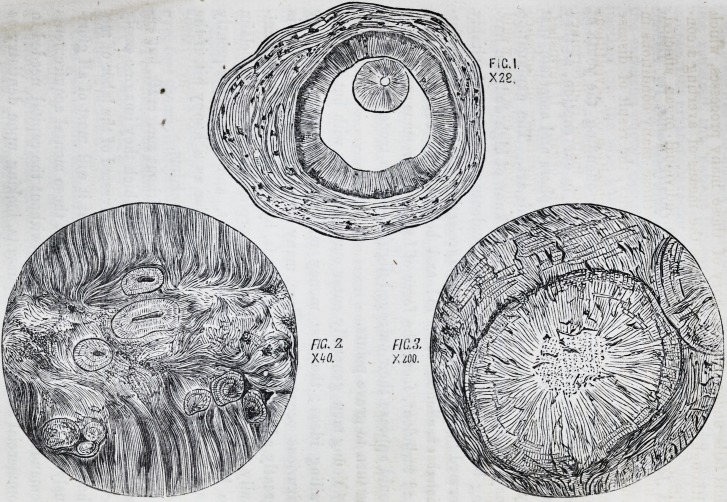# On the Intrinsic Calcification of the Permanent Tooth Pulp, as Constantly Associated with Dental Caries
*The author of this highly interesting paper, having furnished us with a printed copy, we take pleasure in placing it before our readers—omitting, however, the last plate.—Eds.


**Published:** 1856-07

**Authors:** S. James A. Salter


### ARTICLE II.

*On the Intrinsic Calcification of the Permanent Tooth Pulp, as constantly associated with Dental Caries.** By S. James A. Salter, M. B., F. L. S., &c.

My object in this communication is to give an account of a peculiar morbid condition which occurs in the tooth pulp of carious teeth, affecting the pulp after it has arrived at its adult form, and its functions, in the development of primary dentine, have ceased.

This pathological change may be defined as the impregnation of the various tissues of the tooth-pulp with calcareous matter —their calcification, in fact occurring in multitudes of isolated points, and by the multiplication and enlargement of these islands of calcification, involving more and more of the structures of the pulp, and its ultimate conversion, under certain favorable circumstances, into that peculiar form of secondary dentine called *osteodentine.* The occurrence, to some extent, of islands of calcification in the pulps of carious teeth is (as far as my

*The author of this highly interesting paper, having furnished us with a printed copy, we take pleasure in placing it before our readers—omitting, however, the last plate.—Eds.

observations go) as universal as it is unknown.* The complete issue of this process in the evolution of osteodentine is by no means common, and only occurs under certain favorable circumstances, when the process of calcification may continue for a long period, uninterrupted by the laying bare of the pulp cavity.

It will be necessary, before entering upon the details of this subject, to make a few remarks upon the changes which occur in the production of normal dentine, and the manner in which the pulp is reduced to its adult condition, as well as the anatomy of the latter organ; and this will be necessary both as a basis for the explanation of the pathological changes, and also to show the analogies, and at the same time the differences,

* Mr. Tomes was probably acquainted with the *results* of this process when in an advanced stage; but he appears to have confounded the structure produced (osteodentine) with dentine of repair, as well as being wholly unacquainted with the intimate nature of the changes that occur. He remarks, "We see instances of this event of irritation in cases where the pulp is *converted* into dentine opposite a point from which a portion of enamel and of the dentine of the crown, has been removed by wear, by fracture, by the file, or even by caries." (Dental Physiology and Surgery, p. 254.) Mr. Tomes is here evidently speaking of dentine of repair, as may be seen by the figures in his own work, and to which he refers (figs. 32, 84 and 98) as well as by my figures in the Guy's Hospital Reports (On Dentine of Repair, &c., Guy's Hospital Reports, vol. viii, part ii.) *Now here the pulp is not "converted" into dentine.* It is produced simply, as in normal dentine, by the out-growths of the superficial cells, and has no analogy to the change I am describing. Again, Mr. Tomes says, "Indeed, if the pulp of a tooth extracted for caries and subsequent odontalgia be carefully examined, there will, with few exceptions, be found more or less calcification near the point towards which the disease had advanced." (loc. cit.) He probably here speaks of calcification of the puip in an advanced stage, this is very palpable to the unaided senses. The pulp, however, *does not* calcify soonest near the decayed part: on the contrary, the pulp is soonest effected by intrinsic calcification near the extremity of the fang, and the limitary clear layer on the surface is the *last* to calcify. That Mr. Tomes was unacquainted with the early condition of intrinsic pulp calcification, as well as the nature of that process, is sufficiently obvious from the following observations: "By irritability, here, is meant an increased susceptibility to pain, and to morbid action, *unattended with organic change.*" "The most frequent cause of irritability is caries immediately prior to its laying open the cavity." (loc. cit.) Now it was from just such a tooth that the pulp was taken from which fig. 5 was drawn, exhibiting most clearly the "organic change" I am describing.

between the normal process and the pathological changes which I here endeavor to elucidate.

The formative tooth-pulp is a papilla, consisting of numerous blood-vessels and nerves, distributed through multitudes of cells and nuclei, and an immature connective tissue, and covered in by a basement membrane—the whole constituting a form precisely similar to the dentine of the crown of the future tooth.— A series of columnar cells, very similar in appearance to those of columnar epithelium, is found arranged in an even layer upon the surface, covered in only by the basement membrane; from the distal extremities of these cells appear outgrowths, in the form of capillary tubes, and continuous with the cell-wall, and these increase in length by the backward recession of the body of the cells, the tubes being thus prolonged inwards; these constitute the animal basis of the dentinal tubes. The intertubular tissue appears to be formed of a hyaline animal structure, in which no histological elements are indicated.*

It must be recollected that this process is entirely superficial, that it occurs from without inwards; that no other histological elements enter into the formation of the dentine besides the cells described; and that the nerves and blood-vessels recede as the dentine advances. The "*conversion*" theory, which involved the idea of a complete change of the entire pulp-structures into *normal* dentine, is now known to be altogether erroneous; and this it is especially important to remember in relation to the present subject.

The mode in which the animal material is impregnated with the calcareous in primary dentine is very remarkable, and has a certain similarity to the morbid condition I am about to describe. It has been shown by Czermak†, and confirmed and still further elucidated by myself‡, that after the animal mate-

* Kolliker, Handbuch, der Gewebelehre des Menchen, 1852. Also Lent, Ueber die Entwicklung des Zahnbeins und des Schmelzes, in Siebold and Kolliker's Zeitschrift fur Wissenschatliche Zoologie, 1854.

† Czermak, Beitrage zur mkroskopischen Anatomie der menschilichen Zahne, in Siebold and Kolliker's Zeitschrift, 1850.

‡ On certain appearances occurring in dentine dependent on its mode of calcification, in Journal of Microscopical Science, vol. i.

rial of dentine has sketched out its anatomical form, it becomes calcified, not by an even gradual impregnation throughout the whole, nor in any relation to the course of its structure, but in isolated globular patches, by the enlargement and fusion of which the whole is formed into an even coherent mass. And I would further remark—what is of value and interest in reference to the present matter—that the animal material is palpably altered at the time of calcification in physical and chemical characters: it is harder, denser, and contains less water in proportion to the animal solid matter.

I have already alluded to osteodentine as the issue under certain circumstances of the morbid change now under consideration, and I would here observe that osteodentine constitutes one of those peculiar forms of dentine called "secondary," on account of their being after-formations—developed, that is, after the primary system of dentine has been matured. I must add, that the other two forms of secondary dentine, "dentine of repair" (formed by laminæ on the inner surface of the pulp-cavity, as repair and recompense for external wear, decay, or fracture) and "dentine-excrescence" (a nodular growth of dentine arising spontaneously on the interior of the pulp-cavity, and without disease or apparent cause)—that these forms are in all essential particulars the same as primary dentine, formed and calcified in the same way, having contour lines parallel to the surface, and not involving blood-vessels or nerves. The re-

Fig. 1.

Fig. 1.—Diagramatic section of canine tooth, showing the relative position of the three forms of secondary dentine. The tinted parts in the pulp-cavity represents the secondary tissue: the "dentine of repair," fills the summit of the pulp-cavity, and corresponds to the worn exterior surface, the vertical lines on either side indicating the direction of the tubes which limit the external lesion and the internal repair. The "dentine excrescence" is situate on the posterior surface of the pulp-cavity in the fang. The forming "osteodentine" occupies the axis of the pulp.

lation of these three forms* to one another have thought it well to illustrate in a diagramatic outline, fig. 1, representing them in an early stage, and before they have become confused together. The tinted mass at the top of the pulp-cavity represents the dentine of repair, in proportion to the wear at the top of the cusp, and the lines passing from the surface to the margin of this repair-tissue are the dentinal tubes, limiting the external injury and the internal recompense; in the axis of the pulp, the tinted line indicates the forming osteodentine; and the nodule on the side of the pulp-cavity is dentine-excrescence, in a frequent situation, and of a usual form. Though osteodentine is distinct from the other forms of secondary dentine, it is, nevertheless, frequently associated with them, especially with dentine of repair; and the union of the one with the other is the ultimate event of the secondary formations, where they reach entire completion; but this union is the very latest part of the change, and the soft external layer on the surface of the pulp, which separates the forming osteodentine on the one side from the primary dentine and the dentine of repair on the other, is the last to calcify.

My attention was first directed to the peculiar morbid change I am describing by noticing the physical alteration the pulp had undergone in association with caries. Upon opening the pulp cavity of a carious tooth, I had noticed that the pulp was firmer and more coherent than in a natural state; that it did not collapse and readily dry up, but retained its form, and was frequently elastic when bent. It has occasionally happened that in extracting a carious tooth it has broken across at the neck, and the crown has come away, leaving the fangs in the jaw, the pulps of the fangs slipping out of their canals and remaining stiffly bristling from the broken surface of the crown. Such a specimen is represented at fig. 2. It was accidentally obtained in extracting a molar tooth, when, from some malformation of the fangs, probably embracing a mass of bone, the tooth broke across, and the pulps of the fangs came away with the crown,

Fig. 2.

*The different varieties of secondary dentine will be found more particularly discussed in a paper of my own, in the Guy's Hospital Reports, already alluded to, where I believe they were first systematically arranged.

leaving the fangs still embedded in the jaw. In this instance, the pulps were of the firmness, and possessed the elasticity, of whalebone, and much reminded me of that substance. When the calcification is absolutely complete as in the specimen figured in fig. 3,* the whole is perfectly hard and brittle. In every stage of calcification, excepting the very last, the axis of the pulp is the hardest and the exterior more or less soft and pulpy.

FIG. 3.

Until the complete solidification of the pulp, it may readily be torn up with points of needles, uniformly in a longitudinal direction.

The color is also modified by the change, but this depends on the degree of calcification that has taken place. If examined in an early stage, before much earthy matter has been deposited, the pulp is usually found very red, and in a state of obvious hyperæmia, but as the change proceeds, the white calcific deposit reduces the color, and the pulp becomes opaque, and nearly white; and if the change reaches so far as to form osteodentine, then it is of semi-transparent yellow, owing to the obliteration of almost all intervals in the structure, and the formation of a nearly homogeneous mass, containing very few channels or interspaces.

To ascertain the meaning of the changes that have taken place, it is necessary to examine the pulps of teeth (taken from those variously decayed) under the microscope, especially with the aid of chemical reagents. And I may here remark, that in examining a very large number of the pulps of carious teeth, decayed in all degrees, and in every variety of place, I have not once failed to discover, in some degree, the condition I am describing; that is, since I have been acquainted with its optical appearances. If a pulp affected to no very great degree by this

FIG. 2.—Crown of molar tooth ; the semi-calcified pulps remaining attached.

Fig. 2.—Molar tooth with pulp wholly calcified, but still unattached to the primary dentine. The crown of the tooth is removed, that of the calcified pulp remaining. Enlarged about two diameters.

* For the loan of the specimen from which this figure is taken, I am indebted to my friend, Mr. Samuel Cartwright, jun.

intrinsic calcification be viewed by transmitted light, and a magnifying power of fifteen or twenty diameters, it is found preternaturally opaque, especially in the centre: where the calcification is deeper in degree, the opacity is greater, and the limit between the clear edge and the opaque axis more obvious. In fig. 4, is represented one of the pulps taken from the tooth shown at fig. 1, magnified eighteen diameters. The clear margin is

FIG. 4.

unusually broad, and the calcified axis is very marked and exactly defined; the margin of the calcified axis is very opaque and black, while along its absolute centre there is a certain amount of luminosity. In all degrees of calcification, excepting the final change, the outer layer of the pulp is seen to be transparent.

In attempting to view the entire pulp with higher powers—say forty diameters,—its opacity is found so great that little can be made out without previously rendering it clearer by the application of chemical reagents. For this purpose, I have usually employed acetic acid and solution of caustic alkali, but principally the latter. Acetic acid renders the whole very clear, but develops the nuclei and blood-vessels, especially the former, to a degree that causes some confusion, and, by its action on the earthy deposit, produces an evolution of carbonic acid gas, though this occurs to a degree much less than would be imagined. Caustic alkali, however, has no objections; it renders the whole brilliantly clear, exhibiting the calcific deposits, and the nerves,—the latter with great beauty and brilliancy.

In fig. 5, is represented a portion of a tooth-pulp, from the fang of a slightly carious molar tooth, treated with solution of caustic potass, and magnified forty diameters. It exhibits very well the appearances characteristic of the calcific change in an early condition. Running along the pulp are seen very numerous nerves, in bundles of various sizes, with clear intervals

FIG. 4.—One of the pulps from the specimen represented at fig. 2, exhibiting the calcified axis, and uncalcified surface. Magnified 18 diameters.

between them; and scattered throughout the whole promiscuously, excepting for a small space at each margin, are multitudes of small bodies, for the most part of a lenticular form, with very decided and dark boundary outline, lightening off to

FIG. 5.

a brilliant centre; the long axis of each, being uniformly the same as that of the pulp. These are the "calcification islands."*

The appearance of these bodies is very peculiar, not only from their form, but from their extreme darkness of outline—reminding one strongly of oil-globules or bubbles of air in fluid—and arising as in them, from the very different refracting power of the object and the material in which it is placed. So much do these bodies resemble bubbles of air (as seen by low powers,) that at first I concluded they were such, and that their peculiar form was caused by the direction of the tissues in which they were found. But this was a mistake. Upon examining pulps in which a very deep impregnation of calcareous matter has occurred with the same reagent, one finds traces of the original

Fig. 2.—Pulp from the fang of a slightly carious molar tooth, treated with caustic alkali, exhibiting the outer surface uncalcified, and numerous calcification islands in and among the nerves in its axis. Magnified 40 diameters.

* I have thought it well to call these "calcification *islands*" in contradistinction to the other form, "calcification *globules*" (their analogues,) met with in primary, and the other two forms of secondary dentine.

structure more and more obliterated, and the whole field of view is seen to be full of the calcification islands more or less fused together, giving an opaque blackish or clouded appearance, in which little can be made out beyond a general longitudinal direction. The extreme of this condition is represented at fig. 6,

Fig. 6.

taken from the fang of a very highly calcified pulp. Here all residue of previous tissue was lost, and the mass consisted of adherent calcification islands, in which, the adhesion was closest in the longitudinal serial direction, giving a coarse fibrous aspect.

It must be observed that the outer surface of the pulp is never the seat of this process, excepting just at last, when the secondary dentine becomes confounded with the primary; one always sees the outer limit clear and transparent, as long, that is, as the pulp can be readily detached from the cavity. The distribution of these calcified islands through the pulp is liable to some variety, but I have usually found them most abundant in those portions near the extremity of the fang, and in the mass occupying the large chamber in the crown of molar-teeth ; and it is in these regions that we most frequently find the calcification advanced to its complete issue in the production of osteodentine, and that more especially in the former. In the neck and summit of the cusps this change occurs late ; I have one specimen in which the cusps are altogether soft and unchanged, while the central mass is in a state of complete calcification.

I will now proceed to the more precise consideration of these

Fig. 6.—Calcification islands, in the last stage, previous to complete fusion, united longitudinally, but laterally separable. Magnified 200 diameters.

calcification islands, in reference to their *form* and *structure*, their *chemical* and *physical characters*, and their *relation to the tissues among which they are found.*

I have already spoken of these bodies as being lenticular in form, and this is by a great deal their most frequent shape; they are most frequently lenticular or elliptical, occasionally a true oval, and very rarely round. A form which is not unusual is represented at fig. 7 *a;* it is evidently composed of a linear series of islands partially fused together, the lines of union being still distinct, and the whole constituting a long ellipse. Occasionally one finds true cylinders, even throughout, tolerably straight, and with truncated extremities. Sometimes they are found in linear series, but not fused together, as seen in fig. 7 *b*. A very frequent form may be described as club-shaped cylinders, with rounded and very slightly bulbous extremities. They always show a great tendency towards a longitudinal series, and they fuse more firmly end to end than laterally (fig. 6.) The size of the islands varies almost indefinitely, from the smallest perceptible microscopic object to others the $\frac{1}{50}$th of an inch in length; the size, moreover, seems very little to affect, if at all to alter, the forms of the islands, for the same shapes may be recognised, and in about the same proportion, in the smallest as in the largest. When viewed with low power, the outlines of the islands seem tolerably even, but when examined with higher powers, the outline is frequently found irregular, from the fact, that as a rule, all, except the most minute, are composed of numerous smaller ones, more or less fused and adherent together. This compound character is not always seen even in some of tolerable magnitude, but in the largest it is most marked; in these, one sees clearly the outlines of the smaller islands, not only at the edge, but deep in their substance, and by altering the focus, lines of partial and imperfect union are

Fig. 7.

Fig. 7.—Occasional forms of calcification islands. Magnified 200 diameters.

developed, very like those seen in primary dentine, where the globules have imperfectly fused. When perfectly fused, the outlines are lost, and the whole, retaining the original form of the pulp, becomes hard and semi-transparent, as in the specimen represented at fig. 3. I have already remarked that this calcification, up to a certain extent, causes a peculiar stiff elasticity of the entire pulp, and it is remarkable that this physical character, general through the whole pulp, should be produced by the distribution of isolated particles of a very different physical nature. The calcified islands are individually perfectly hard and unyielding, and break with brittleness when subjected to much pressure.

Chemically, these ossified masses consist of an animal basis and earthy salt, soluble in muriatic acid, and are probably analogous to ordinary dentine in this respect. When treated with acetic acid they are not much acted upon, but yield only a portion of their salts with the evolution of gas, which appears in the form of air-bubbles among the fluid of the specimen. When treated with hydrochloric acid the action is far more energetic, the whole of the earthy matter appears to dissolve out, leaving a peculiar cartilage-like residue of much firmness, and retaining the exact form of the original island, neither more nor less, it is clearish, and altogether destitute of the dark, highly-refracted outline visible in the object previous to decalcification. I have never succeeded in making out, either in the original or the decalcified islands, any laminæ, tubes, or other histological forms; but this would necessarily arise from the peculiar optical dificulties that exist; a small, isolated, very dense mass among tissues of great tenuity would scarcely yield indications of its own structure.

The relation of these islands of calcification to the tissues in which they are found is the most remarkable feature in this subject, and is likely to be of no small physiological value in reference to the whole subject of calcification of soft structures. I have already enforced the fact that the calcification (by globules) of primary dentine and the other two forms of secondary dentine takes place in a soft tissue formed from particular cells, and normally adapted and intended as the recipients of such impregnation; but in the structure we are now considering, the case is far otherwise; here we have an organ, the tooth-pulp consisting, besides cells and nuclei, of connective tissue, blood-vessels, and multitudes of nerves, and we find the whole of these structures swallowed up and obliterated by the calcification process. I have just stated that by the removal of the earthy matter with hydrochloric acid a form of animal matter identical with the previous calcification island is left, and this is the case with the smallest as well as the largest: *the addition of the earthy matter is in noway interstitial.*

FIG. 8

FIG. 9.

The best power to examine these bodies in relation to the other tissues is that magnifying 200 diameters; and for this purpose the application of caustic alkali is a very necessary preliminary step.

It is found almost impossible to isolate the calcification islands from the tissues in which they are found; and their intimate relation with the nerves is very remarkable, and easily de-

Fig. 8.—Calcification islands in the body of a compound nerve; the nerve-fibres in no way displaced or bulged out by their presence. Magnified 200 diameters.

Fig. 9.—Calcification island (produced apparently by the fusion in linear series of three smaller ones) in a compound nerve, from the pulp of a carious tooth; a single nerve-fibre being, as it seemed, involved in the calcareous particle.* Magnified 200 diameters.

* The author regrets that this figure has not been accurately rendered by the artist; the calcification island is on a plane too superficial, and the course of the central nerve-fibre into it is not so clear as in the original.

monstrated, from the fortuitous circumstance that the reagent necessary to clarify the object renders the nerve structures only more distinctly visible.

The islands of calcification are very numerous among the nerves, and are seen on the edge of bundles of nerves, and in all parts of the axes. The nerve-fibres do not seem in any way pushed aside by their presence, for we see them of considerable size in the axis of a compound nerve without any bulging of its surface or edges (fig. 8). The relation of the individual nerve-fibres it is not so easy to demonstrate; still (though I am not disposed to commit myself to the unqualified statement,) I believe I have frequently seen a single fibre pass into the very axis of the calcification island on one side and leave it on the other, being lost and obliterated for that space in its course. Such a specimen I have figured (fig. 9,) and the only source of fallacy one can imagine might arise from the nerve passing below the calcification island on a different plane, and so be covered up by it; but this I do not believe. What is more unmistakeable, and convinces me that the nerves do really sustain the calcific impregnation, is what is seen in the advanced stages of that change. One very frequently sees a considerable bundle of nerves, entering, so to speak, a dense semi-fused mass of calcification islands and leaving them further on in their course, the nerves having the ordinary and normal structure up to and in contact with the calcified mass, which obviously holds exactly the position previously held by the intermediate portion of the compound nerve. What has become of the compound nerve for that interval, unless impregnated and obliterated by the calcification process? I can conceive no other explanation of these appearances than by imagining that the change pervades the tissues promiscuously and without elective choice. I do not consider that the inability to demonstrate nerves in the decalcified mass in any way militates against this doctrine; the calcification process is not merely an addition of earthy salts to the animal mass; the latter is altered at the same time.*

*Kölliker, while speaking of the evidences to be obtained from the examination of decalcified bone, remarks, "that everything which thus presents itself,

Frequently, though the calcification be far advanced, a single nerve-fibre can be traced for a considerable distance; the same with small bundles, but never, as far as I have seen, with large.

The cells and nuclei, which are seen pretty numerous in the substance of the adult pulp among its vessels and nerves, undoubtedly share in the general change, and probably the former produce the few dentinal tubes that are found in osteodentine.

Of the change produced in the blood-vessels I cannot speak with much accuracy, but they seem to share, to a certain extent, in the general change. If we examine a section of perfectly formed osteodentine (pl. i, fig. 2), the few blood-vessels in the axes of the dentine haversian systems are seen certainly much less numerous than those in the original pulp, and even some of these, as I shall presently show (pl. i, fig 3), become obliterated by calcification. I have, however, frequently seen them, of quite healthy structure, when almost every trace of the other tissues has been lost. Upon treating a tooth pulp with rather dilute acetic acid, I have followed the vessels among the calcified masses for a considerable distance, their coats exhibiting the characteristic nuclei with unmistakeable clearness, and quite unaltered. It seems that the blood-vessels, the larger ones at least, undergo the calcific change at a late period.

We trace the process of calcification readily enough up to a certain point—where the islands are still separable and not wholly fused together: we readily pick abroad small fragments, and examine them as moist specimens: it would be physically impossible to make sections for dry mounting, and we require, moreover, some clarifying reagents to make the specimen intelligible. When, however, the calcification is absolutely complete, we find a firm coherent mass, capable of being reduced to sections as thin as normal dentine, and displaying appearances which are recognized as osteodentine.

in an isolated form, is not necessarily a morphological unity." The converse of this may be said with equal truth—that because a hystological element, which is supposed to enter into the animal basis of a calcified mass, cannot be isolated from the same decalcified, it does not prove the original non-existence of that tissue.

It is not my intention to give any detailed description of the anatomy of osteodentine, as it is so generally understood and described in works of anatomy, &c. There are, however, some points in its structure which have never been noticed, and which are of interest, not only for themselves, but as throwing light upon, and being explained by the changes I have been describing.

Osteodentine may exist in any number of systems, and the amount of pulps involved does not appear to affect the maturity at which a partial calcification may arrive. In fig. 1, pl. i, is represented a section made near the apex of the fang of a carious bicuspid tooth, containing one, and only one, perfectly formed dentine system, the rest of the pulp not being affected.*

Osteodentine has fewer tubes than any other form of dentine, and is consequently very transparent: this transparency does not altogether arise (as does that of dentine of repair) from the filling up of the tubes with secondary deposit within them; many of the tubes are doubtless so filled up, as is the case with all dentine formed in states of tooth irritation or inflammation, but they are nevertheless really less abundant, and that is the true cause of the peculiar transparency. This circumstance is quite intelligible upon the idea that all the tissues of the pulp share alike in the common change: in this case doubtless the calcified nerves and blood-vessels and connective tissue would not develop tubes, but only those elements of the pulp which are similar to the cells on the surface of the pulp, constituting the "membrana eboris."

Osteodentine is described as consisting of systems of dentine around isolated blood-vessels; and so it generally is, but occasionally, and not very infrequently, indeed, the central canal can no longer be seen; it is obliterated by the calcification of the blood-vessel, and its position occupied by an indefinite clear structure. Here the last of the soft tissues of the pulp is swallowed up by the calcific change. (Plate i, fig. 3.)

Again, a tissue resembling dentine is not the only result of

* For this specimen, I am indebted to the kindness of my friend, Mr. Walter Jones, of Worcester.

this process. Under certain peculiar circumstances, I have found the pulp converted into crusta petrosa: the peculiar circumstances which appear to be connected with this change being a preternaturally abundant communication between the pulp and the periosteum—the communication being large and short, so that the pulp and the periosteum are almost continuous. I have* elsewhere exhibited specimens illustrative of this circumstance, without, on that occasion, drawing any general inferences from them. In these instances, by means of erratic vascular canals, the communication between the pulp and the periosteum was very direct and abundant, and in each there was a development of bone in the cavity. A still more remarkable example of this condition I found in a carious temporary molar tooth, which had been retained in the mouth till eighteen years of age. In this instance, the fangs had been somewhat absorbed, especially on the inner surfaces, so as to lay open the pulp cavities to near the main chamber of the tooth, and moreover the canals were considerably enlarged; by this means the pulp and the periosteum were almost as one. Upon making a section of this tooth across the crown at the line indicated in the figure of it, the pulp was found converted into a mass of crusta petrosa and dentine confounded together: there were many vascular canals among it, but these were mostly ground out in the process of making the specimen: they appeared to communicate with the principal one which is still visible. The laminæ were numerous, and of the character of those found in ordinary cement; the dentinal tubes were tolerably, though not very abundant, and the two tissues were entirely confounded together. There were also numerous interspaces among the tissue, the result of the imperfect fusion of the calcification islands.

These observations on the calcification of the tooth pulp, and the particular relation of the calcific change to the tissues of that organ, are not without value in a practical sense, mutually

* "On Erratic Vascular Canals in Teeth, associated with the Development of Bone in the Pulp-cavity." ("Trans. Path. Soc.," vol. v.)

explaining, and being confirmed by certain circumstances which arise in operations on the teeth.

There are two operations connected with the teeth, in one of which constantly, and in the other frequently, we have to do with the pulp cavity, and are influenced in our proceedings by the condition of the pulp itself. These are respectively *tooth-pivoting* and *stopping* or *plugging*.

In tooth-pivoting, a gold or other pin is introduced into the pulp-cavity of single fanged front upper teeth, with the crown of an artificial tooth attached to it, after the crown of the natural one has been cut off for unsightly caries. But before adapting the artificial tooth it is necessary to prepare the pulp-cavity by means of a drill for the reception of the pin. In this process we have to do with the pulp in every varied stage of calcification, and the vital phenomena which it exhibits are exactly in accordance with the degree of change. Where the calcification is slight, the pulp is exquisitely sensitive, and the application of the drill produces intense pain; and in this condition it bleeds. As the calcification advances, the sensibility and the tendency to bleed gradually and regularly subside, till at length in the completely formed osteodentine these indications of vitality have ceased altogether, and the calcified pulp may be drilled with as little inconvenience as attends the cutting of a hair or nail. Sometimes, however, one finds, even in an advanced stage of calcification, an intensely sensitive spot in the pulp, and sometimes the point of the drill will be stained with blood in piercing it; but this is easily explained, by imagining that in the one case a nerve-fibre, and in the other a blood-vessel has escaped the common change. Still it must be stated, as a rule, that *the sensibility and the bleeding are inversely as the calcification.*

In plugging teeth it is necessary to cut away all the softened and carious dentine, and we not unfrequently reach the calcified pulps, when all the phenomena I have just described similarly display themselves.

The calcification process I have now been describing must certainly be considered as a morbid change,* though in effect a reparative one; it occurs in disease and as its result, but when complete, obviates the ill effects which would ensue. It thus falls under the category of those many processes, which although essentially morbid, are the means of averting a condition which would be fatal to the individual organ affected. This process finds its counterpart in those many conditions in which irritation and increased vascularity, the result of disease in contiguous structures, issue in the deposition of adventitious matter: in this instance the adventitious matter is determined by the normal nutritional affinities of the organ. It furnishes, moreover, an interesting example of a qualitative disturbance of nutrition resulting from a vascular disturbance which would seem to be merely quantitative.

* I cannot think that this change in a diseased condition in man is quite analogous to what we find in some of the lower animals in which osteodentine is normally formed. In them it uniformly commences at the upper extremity of the pulp-cavity, and is adherent to the primary dentine. One can easily conceive that the pulp, in these cases, breaks up into compound papillæ, that on the *surface* of these the dentine is formed, while the nerves and vessels recede before it.

### PLATE I.

Fig. 1. Transverse section of a bicuspid tooth, near the apex of the fang, with a single system of osteodentine, and a large patent pulp cavity. (Magnified 28 diameters.)

Fig. 2. Section of fang of carious tooth: the pulp-cavity completely filled with osteodentine. (Magnified 40 diameters.)

Fig. 3. A single system of osteodentine, in which the central vascular canal is obliterated by calcification. (Magnified 200 diameters.)

FIG. 1. X 28.

FIG. 3. X 200.

FIG. 2. X 40.

## Figures and Tables

**Fig. 1. f1:**
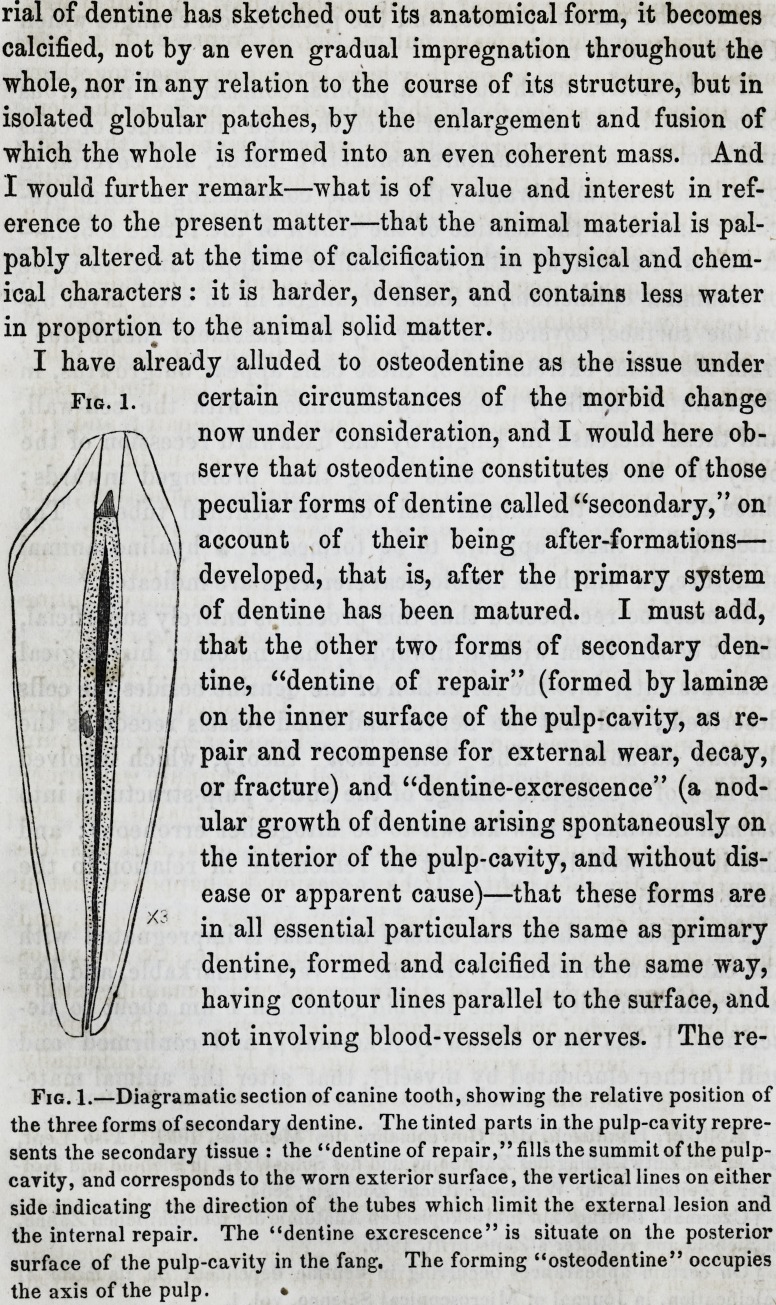


**Fig. 2. f2:**
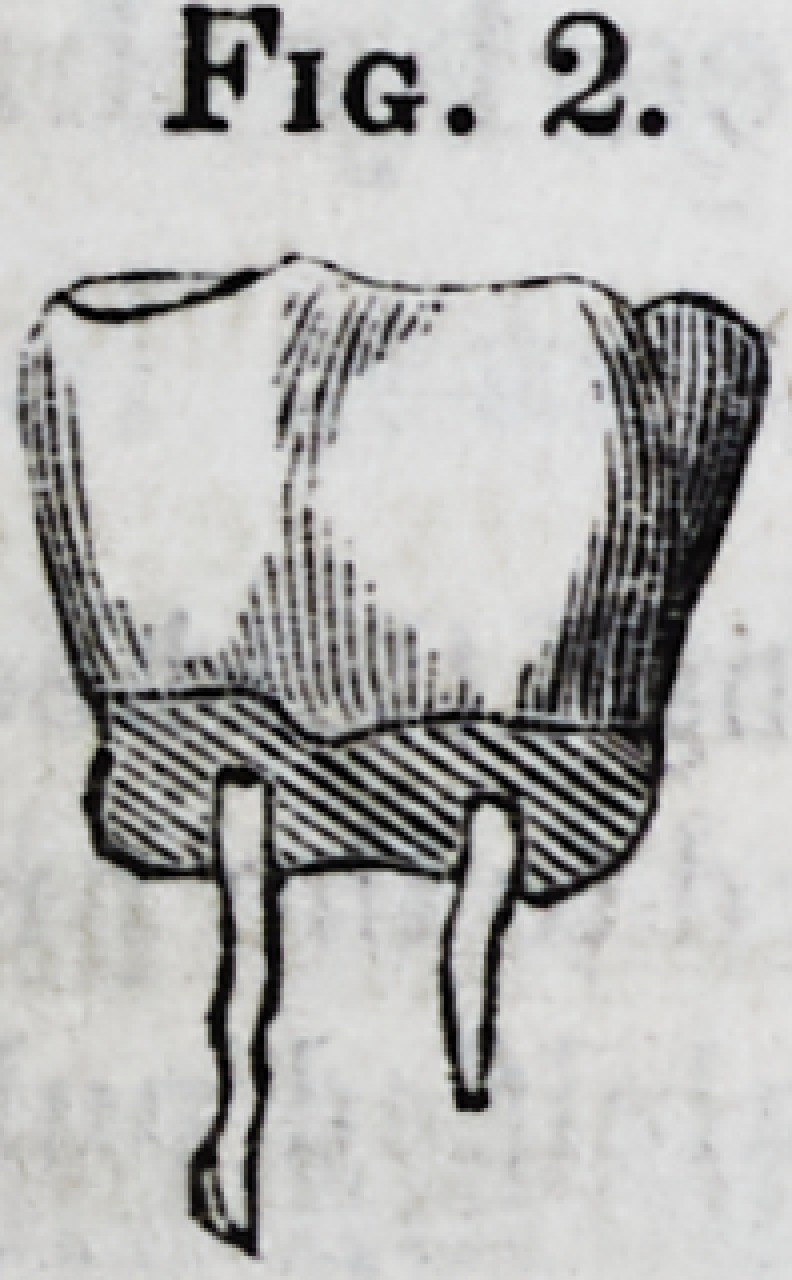


**Fig. 3. f3:**
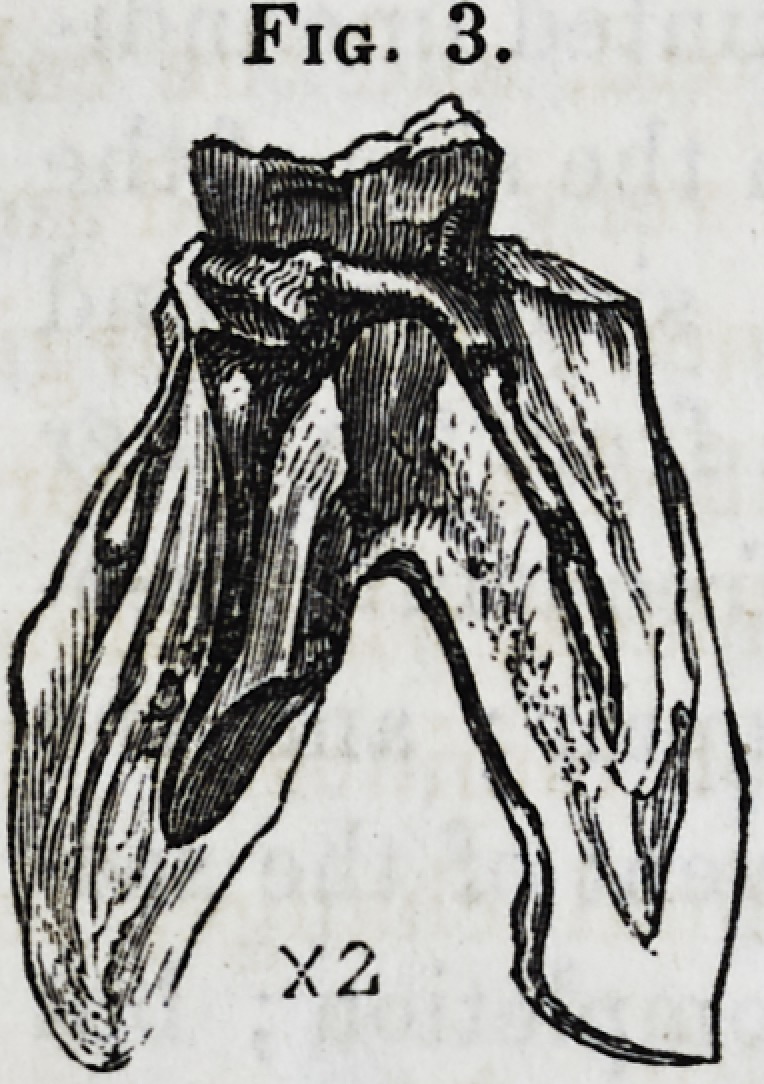


**Fig. 4. f4:**
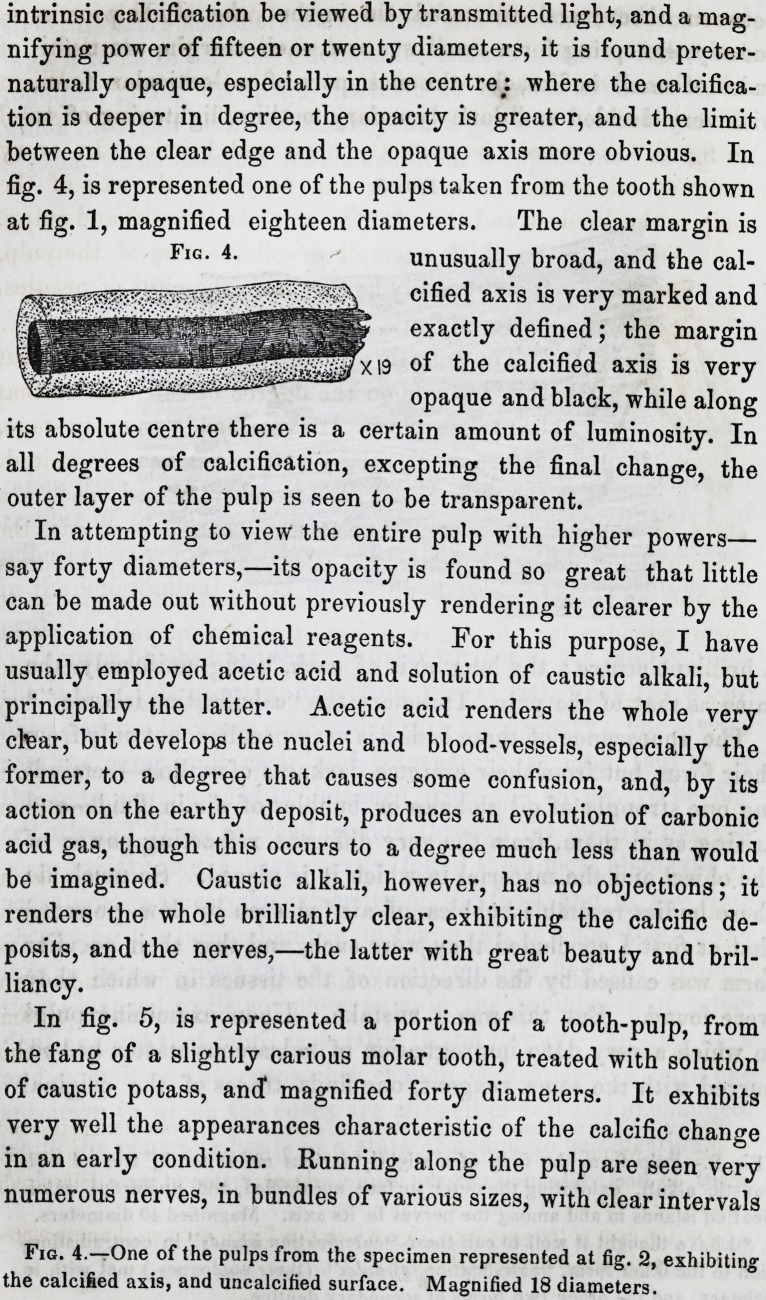


**Fig. 5. f5:**
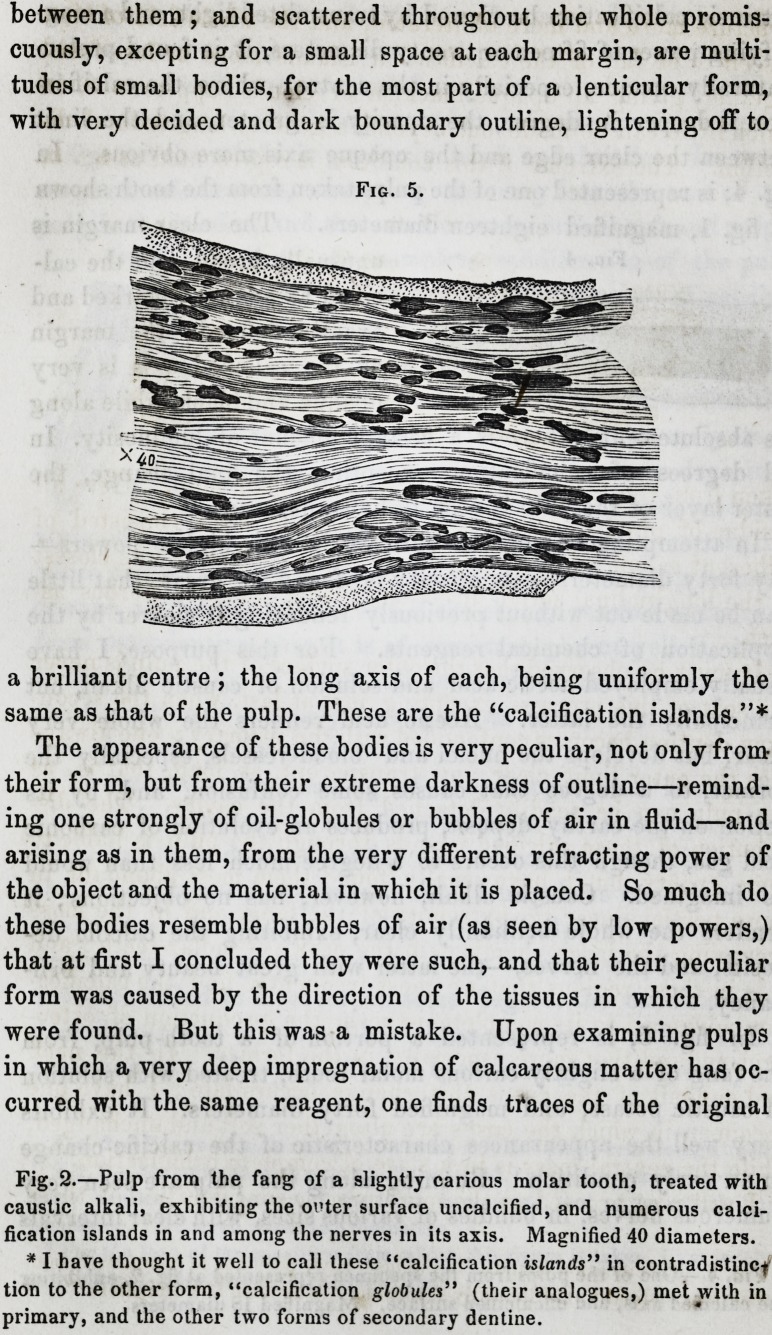


**Fig. 6. f6:**
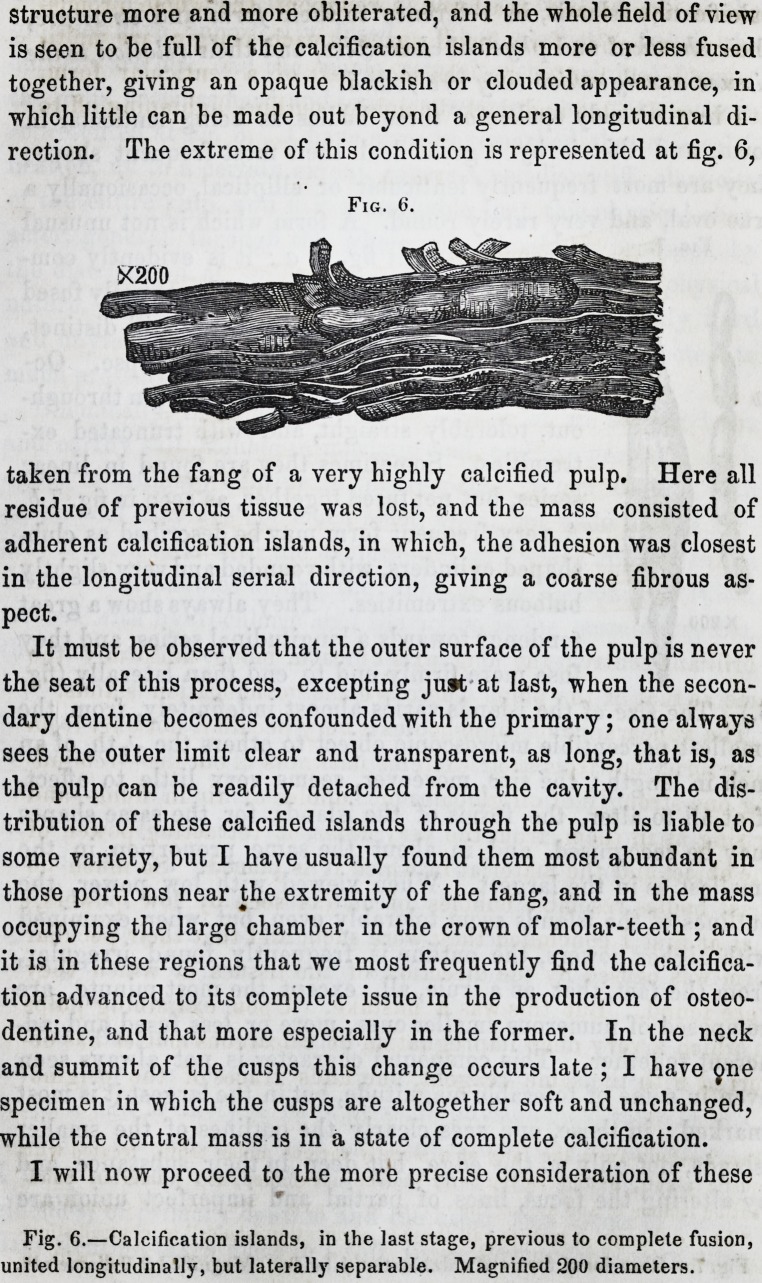


**Fig. 7. f7:**
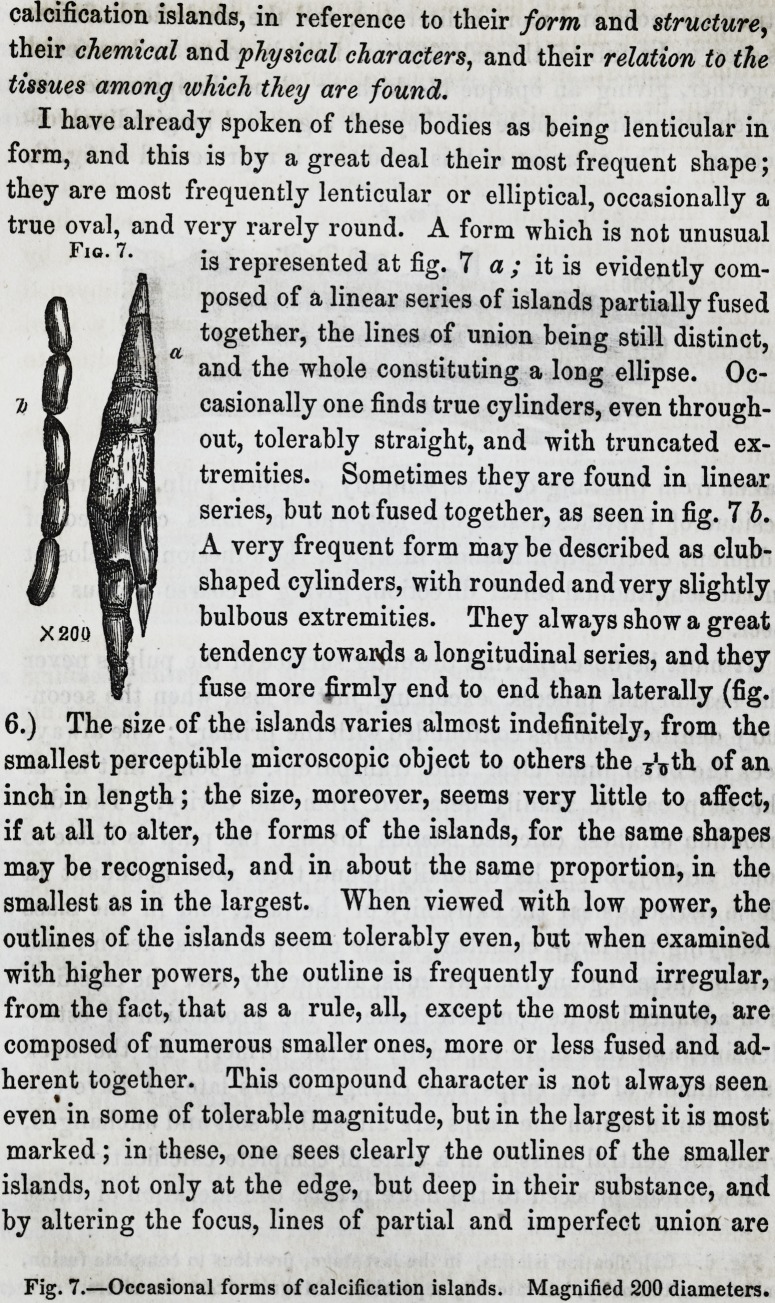


**Figure f8:**
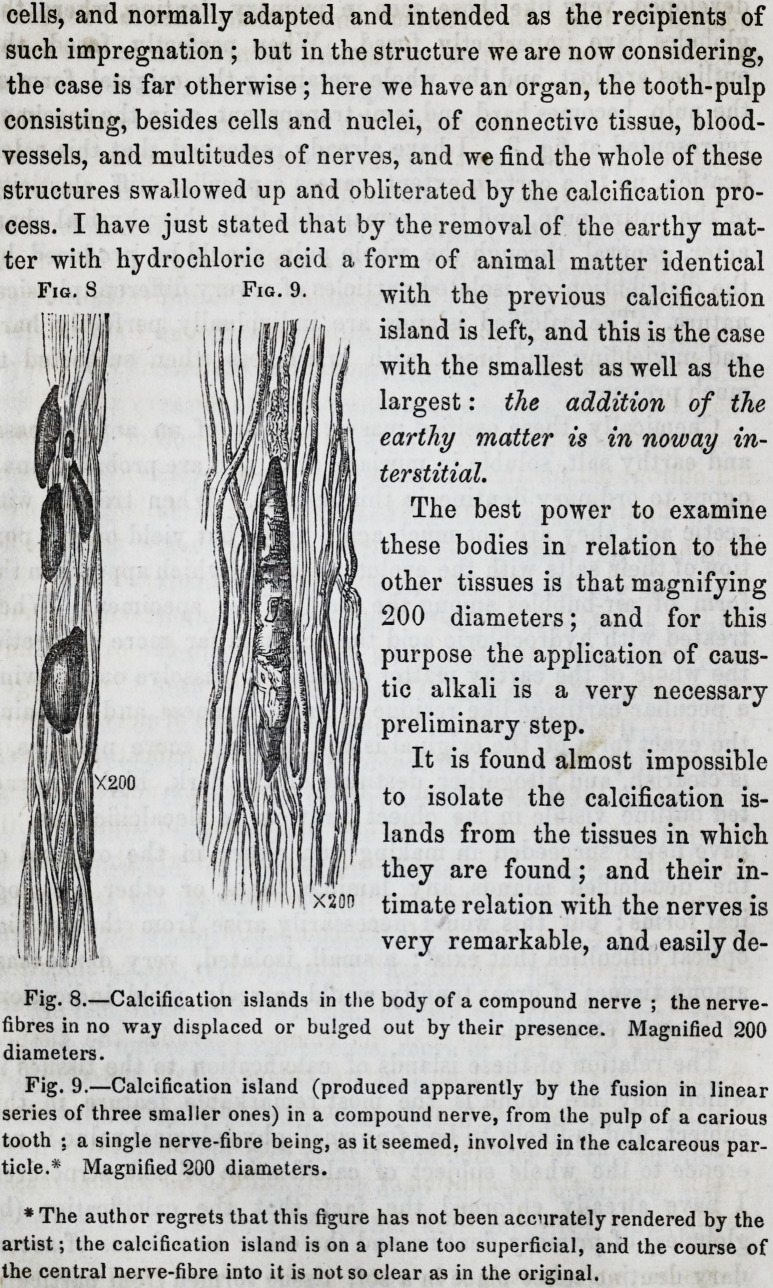


**Figure f9:**